# Interplay between PCBP2 and miRNA modulates *ARHGDIA* expression and function in glioma migration and invasion

**DOI:** 10.18632/oncotarget.6869

**Published:** 2016-01-09

**Authors:** Xihua Lin, Bin Yang, Wei Liu, Xiaochao Tan, Fan Wu, Peishan Hu, Tao Jiang, Zhaoshi Bao, Jiangang Yuan, Boqin Qiang, Xiaozhong Peng, Wei Han

**Affiliations:** ^1^ Institute of Basic Medical Sciences Chinese Academy of Medical Sciences, School of Basic Medicine Peking Union Medical College, Beijing, China; ^2^ Department of Neurosurgery, Beijing Tiantan Hospital, Capital Medical University, Beijing, China; ^3^ Department of Thoracic/Head and Neck Medical Oncology, University of Texas MD Anderson Cancer Center, Houston, Texas, USA

**Keywords:** PCBP2, ARHGDIA, miRNA, glioma, migration and invasion

## Abstract

RNA-RNA and protein-RNA interactions are essential for post-transcriptional regulationin normal development and may be deregulated in cancer initiation and progression. The RNA-binding protein PCBP2, an oncogenic protein in human malignant gliomas, is an essential regulator of mRNA and miRNA biogenesis, stability and activity. Here, we identified Rho GDP dissociation inhibitor α (ARHGDIA) as a target mRNA that binds to PCBP2, and we uncovered the role of ARHGDIA as a putative metastasis suppressor through analyses of *in vitro* and *in vivo* models of EMT and metastasis. Furthermore, we demonstrated that ARHGDIA is a potential target of miR-151-5p and miR-16 in gliomas. The interaction between PCBP2 and the 3′UTR of the ARHGDIA mRNA may induce a local change in RNA structure that favors subsequent binding of miR-151-5p and miR-16, thus leading to the suppression of ARHGDIA expression. PCBP2 may facilitate miR-151-5p and miR-16 promotion of glioma cell migration and invasion through mitigating the function of ARHGDIA.

## INTRODUCTION

The Rho GDP dissociation inhibitor α (RhoGDIα), also known as ARHGDIA, RhoGDIA, or RhoGDI1, is thought to be a negative regulator of Rho family G-proteins, namely Rho GTPases [1]. Currently, three evolutionarily conserved mammalian RhoGDIs have been identified: RhoGDIA (α), RhoGDIB (β) and RhoGDIG (γ). Of the three isoforms of RhoGDI identified to date, ARHGDIA is ubiquitously expressed and interacts with all three key Rho GTPases, including RhoA, Cdc42 and Rac [2]. RhoGDIs bind to Rho GTPases and maintain them in a biologically inactive state in the cytoplasm, which affects the regulation of actin cytoskeleton processes, including formation of focal adhesions, stress fibers, lamellipodia and filopodia; membrane ruffling and cell motility; and changes in cell morphology [3].

ARHGDIA expression levels are altered in different types of cancers. For example, ARHGDIA expression is increased in colorectal and ovarian cancer [4, 5]. In contrast, ARHGDIA expression is reduced in brain cancers and hepatocellular carcinoma [6, 7]. In breast cancers, conflicting results have been reported, with ARHGDIA expression being increased or decreased in different studies [8-10]. Loss of ARHGDIA enhances metastasis and resistance to tamoxifen in breast cancer [11] and promotes the development and progression of prostate cancer [12]. A recent article has reported that ARHGDIA is decreased in hepatocellular carcinoma, and its expression is regulated in part by the recently characterized miR-151-5p, which is located in an intron of the gene encoding focal adhesion kinase (FAK) and is expressed with FAK. [7]. However, the regulatory mechanism of ARHGDIA suppression of tumor migration and invasion remains largely unexplored. Accumulating evidence from experimental and clinical studies also suggests that EMT activates tumor migration and invasion by endowing cells with a more motile, invasive phenotype [13-15].

Our previous study has shown that the RNA-binding protein PCBP2 is increased in human glioma tissues and cell lines. Knockdown of PCBP2 inhibits glioma growth *in vitro* and *in vivo* through inhibiting cell cycle progression and inducing apoptosis. ARHGDIA has been identified as a target mRNA binding to PCBP2 from RIP-Chip and biotin pull-down analyses [16]. This result prompted us to investigate the expression pattern and function of ARHGDIA in gliomas and the role of PCBP2 in this process. It is becoming apparent that RBPs affect the biogenesis, activity and stability of miRNAs, which have been shown to be involved in normal development and cancer by an enormous body of evidence [17-22]. For example, the RBPs Pumilios are required for miR-221/miR-222-mediated repression of the p27 tumor suppressor. The binding of PUM1 induces a local conformational change in the *p27* transcript that exposes a miR-221/miR-222-binding site [23]. An RNA-binding protein called Dead end (Dnd1) inhibits the function of several miRNAs by blocking the accessibility of target mRNAs [24]. This indicates the existence of interplay between RBPs and miRNAs that correlates with gene expression and processes.

Here, we show that the *expression* of ARHGDIA is *frequently decreased* in high-grade malignant gliomas. We uncovered the role for ARHGDIA as a putative metastasis suppressor through analyses of various *in vitro* and *in vivo* models of EMT and metastasis. Furthermore, we demonstrated that ARHGDIA is a potential target of miR-151-5p and miR-16 in gliomas, and PCBP2 binding of the ARHGDIA-3′UTR induces a local change in RNA structure that favors association with miR-151-5p/miR-16, efficiently suppressing ARHGDIA expression, which may strongly affect tumor growth, migration, and invasion. Our findings uncover a novel RBP-induced structural switch modulating miRNA-mediated gene expression regulation.

## RESULTS

### ARHGDIA protein but not mRNA is frequently downregulated in gliomas

**Figure 1:**
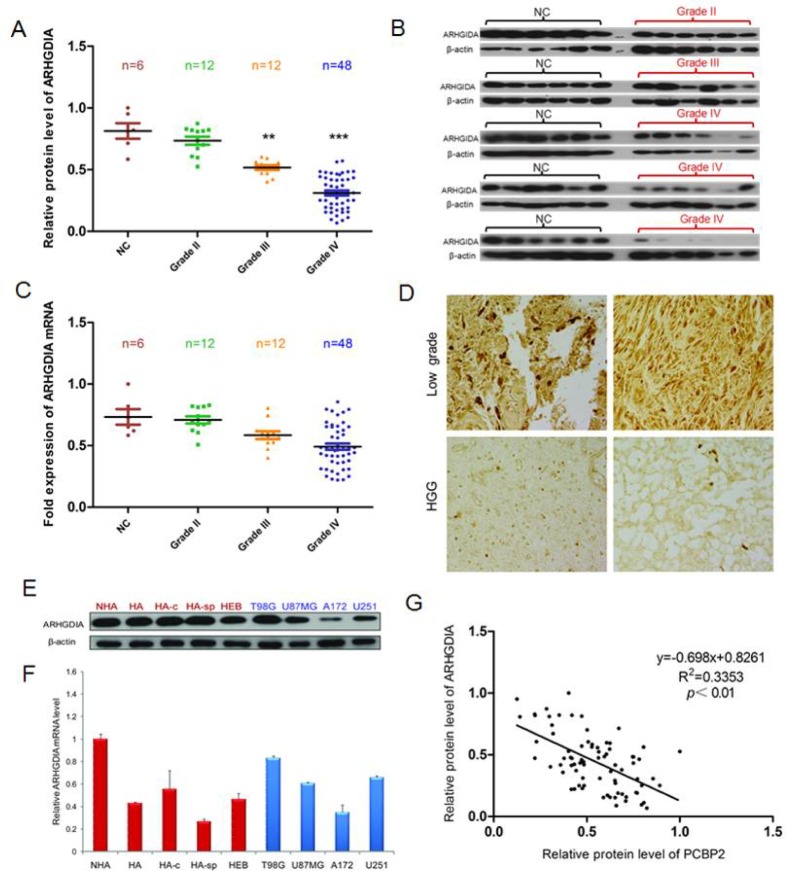
ARHGDIA protein but not mRNA is frequently downregulated in glioma tissues compared to control brain tissues **A**. Relative ARHGDIA protein levels in 6 control brain tissues,12×II grade, 12×III grade, and 48×IV glioma tissues. **B**. Representative western blot showing ARHGDIA protein levels in 6 control brain tissues, 6×II grade, 6×III grade, and 48×IV glioma tissues. β-actin was used as a loading control. **C**. Real-time PCR analysis of relative ARHGDIA mRNA expression in 6 control brain tissues, 12×II grade, 12×III grade, and 48×IV glioma tissues. GAPDH was used as a control. n.s., nonsignificant. **D**. Representative immunohistochemical staining of ARHGDIA in 16 low-grade glioma tissues and 19 high-grade glioma tissues using anti-human ARHGDIA antibodies. Original magnification,×400. **E**. Western blotting showed ARHGDIA protein levels in 4 human astrocyte cell lines (HA, NHA, HA-c, HA-sp), 1 human embryonic brain cell line (HEB), and 4 glioma cell lines (T98G, U87 MG, A172, U251). **F**. Real-time PCR analysis of ARHGDIA mRNA in 4 human astrocyte lines, 1 human embryonic brain cell line and the 4 indicated glioma cell lines. **G**. Relative ARHGDIA and PCBP2 protein levels in 6 control brain tissues,12×II grade, 12×III grade, and 48×IV glioma tissues.

To examine the expression pattern of ARHGDIA in gliomas, western blotting and real-time PCR were performed to analyze the gene expression profiles. Total RNA and proteins were extracted from 6 brain tissue samples from donors who were not diagnosed with gliomas (normal control brain tissues, NC) and compared with RNA and proteins from 72 glioma tissue samples, which consisted of 12 grade II, 12 grade III and 48 grade IV glioma tissues. Strong expression of the ARHGDIA protein but not mRNA was found in the 6 control brain tissues, but there was a clear loss of ARHGDIA in grade III and grade IV glioma tissues. Gradually weaker ARHGDIA expression was found from grade III samples to grade IV samples (Figure 1A-1C). Immunohistochemical analysis of ARHGDIA was conducted using paraffin sections of low-grade glioma tissues (n=16) and high-grade glioma (n=19) tissues from 35 patients, and the results showed that ARHGDIA expression was decreased in the high-grade glioma samples compared with the low-grade glioma samples (Figure 1D). The results showed that the protein level but not mRNA expression of ARHGDIA was frequently downregulated in glioma tissues compared with control brain tissues. Additionally, we also measured mRNA and protein levels of ARHGDIA in 4 normal human astrocyte cell lines (HA, NHA, HA-c and HA-sp), 1 human embryonic brain cell line (HEB) and 4 different human glioma cell lines (T98G, U87 MG, A172 and U251). Moderate to high expression of ARHGDIA was detected in all cell lines (Figure 1E, 1F). We also analyzed the relative protein expression of ARHGDIA and PCBP2 in the above-mentioned glioma tissues. The protein level of ARHGDIA was negatively associated with the protein level of PCBP2 by Pearson's correlation analysis, with statistical significance established at *P*<0.05 (Figure 1G). The glioma patients' clinical information is shown in Supplementary Table 1.

### Overexpression of ARHGDIA inhibits glioma migration and invasion *in vitro* and *in vivo*

**Figure 2:**
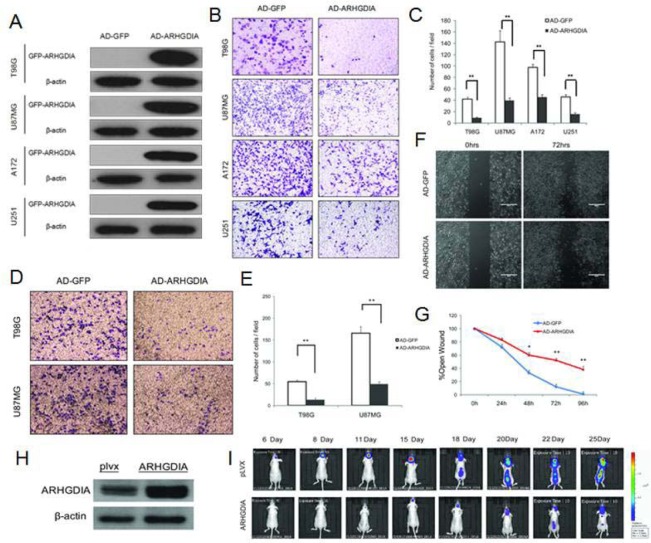
Overexpression of ARHGDIA inhibits glioma migration and invasion **A**. Western blot of GFP-ARHGDIA expression in 4 glioma cell lines (T98G, U87 MG, A172, U251) infected with AD-GFP and AD-ARHGDIA for 48 h. (**B**., **C**.) Transwell migration assays of 4 glioma cell lines (T98G, U87 MG, A172, U251) were performed after infection with AD-GFP and AD-ARHGDIA for 48-72 h. The results are representative of at least three independent experiments. Graphs indicate the average number of cells per field of the indicated cell lines in migration assays. Data show the mean ± SD, *P<0.05,**P<0.01. (**D**., **E**.) Invasion assays of 2 glioma cell lines (T98G, U87 MG) were performed after infection with AD-GFP and AD-ARHGDIA for 48-72 h. The results are representative of at least three independent experiments. Graphs indicate the average number of cells per field of the indicated cell lines in invasion assays. (**F**., **G**.) Wound-healing assays in T98G cells were performed after infection with AD-GFP and AD-ARHGDIA for 0-96 h. Pictures were taken every 24 h. The graph represents the width of the remaining open wound calculated in relation to time 0. (H) Western blot of ARHGDIA expression in plvx-U87 MG and ARHGDIA-U87 MG stable cells for 72 h. **I**. plvx-U87 MG and ARHGDIA-U87 MG stable transfectants (5×10^5^) were injected intracranially into 10 nude mice and then imaged from day 6 to day 25. Tumor sizes were quantified by *in vivo* bioluminescence imaging (BLI) in experimental intracranial metastasis models of gliomas.

To investigate the biological function of ARHGDIA in gliomas, we constructed a recombinant adenovirus encoding a green fluorescent (GFP) fusion protein—GFP-ARHGDIA protein (AD-ARHGDIA, 55 kDa)—and used the recombinant adenovirus encoding GFP protein (AD-GFP, 28 kDa) as a negative control. After the adenoviral transduction for 48 to 72 hours in 4 glioma cell lines (T98G, U87 MG, A172 and U251), western blotting analysis confirmed that a high level of ARHGDIA protein expression was achieved in infected cells compared with the negative control-infected cells (Figure 2A). Then, transwell migration assays were performed in 4 glioma cell lines (T98G, U87MG, A172 and U251), and 2 glioma cell lines (T98G, U87 MG) were chosen for use in the matrix invasion assays, which were more invasive and had faster wound healing than other two glioma cell lines (A172 and U251) and were thus more suitable for the matrix invasion assays. The results demonstrated that overexpression of ARHGDIA dramatically inhibits both migration and invasion of the glioma cells (Figure 2B-2E). Strikingly, in the wound-healing assay performed with the T98G cell line, we found that AD-ARHGDIA overexpressing cells were more proficient than AD-GFP transduced cells at closing an artificial wound created over a confluent monolayer (48 h, *P<0.05, 72 h, 96 h,**P<0.01) (Figure 2F, 2G).

To test the effect of increased ARHGDIA in glioma cells *in vivo*, we constructed an ARHGDIA-luc-expressing lentivirus (lenti-ARHGDIA) and control lentivirus (lenti-plvx), and we established stable cell lines, denoted human glioblastoma U87 MG, after lenti-ARHGDIA transduction (ARHGDIA-U87 MG) and lenti-plvx transduction (plvx-U87 MG). Intracranial orthotopic xenografts were established by implanting approximately 5 × 10^5^ U87 MG-Luc cells stably expressing either ARHGDIA (ARHGDIA-U87 MG) or the control lentivirus (plvx-U87 MG) intracranially (Figure 2H). Tumor sizes and intracranial invasion were quantified and observed by bioluminescence imaging from day 6 to day 25. Overexpression of ARHGDIA significantly inhibited tumor size and intracranial invasion (Figure 2I).

### Overexpression of ARHGDIA reverses mesenchymal-like characteristics of glioma cells

**Figure 3:**
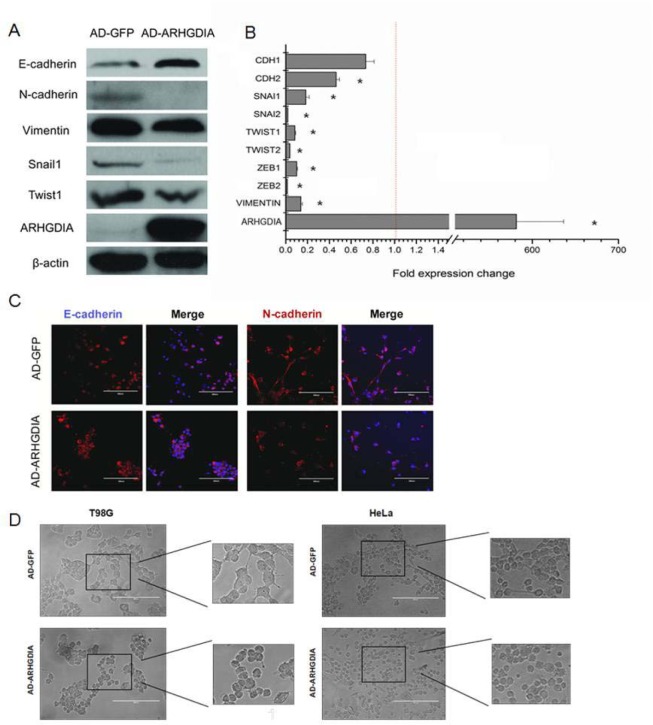
Overexpression of ARHGDIA reverses mesenchymal-like characteristics of glioma cells **A**. Immunoblot showing the downregulation of mesenchymal markers and upregulation of epithelial markers after ARHGDIA overexpression in T98G cells infected with AD-GFP and AD-ARHGDIA. **B**. Quantitative real-time PCR analysis of ARHGDIA and EMT-related genes in T98G cells infected with AD-GFP and AD-ARHGDIA. Real-time PCR analysis values were normalized to GAPDH. Experiments were performed three times, and data show the mean ± SD,*P<0.05,**P<0.01 by Student's *t-*test. **C**. Immunofluorescence analysis of E-cadherin (red) and N-cadherin (red) merged with nuclear DAPI staining (blue) in AD-GFP- and AD-ARHGDIA-infected T98G cells. The scale bars represent 200 μm. **D**. Morphological changes of HeLa cells and T98G cells after ARHGDIA overexpression. Scale bar, 200 μm.

The epithelial-mesenchymal transition (EMT) is a complex process that occurs in cancer migration and invasion. To evaluate whether overexpression of ARHGDIA inhibited EMT-related changes, we first assessed the EMT-related genes after infection with AD-GFP and AD-ARHGDIA using western blotting analysis in the T98G cell line, which is the most commonly used cell line in glioma research. The cells showed significant increases in epithelial marker E-cadherin expression and reductions in mesenchymal markers N-cadherin and vimentin expression, as well as reductions in EMT-related transcription factors, such as Snail1 and Twist1 (Figure 3A).We further found that enforced ARHGDIA expression in T98G cells downregulated key transcriptional factors such as SNAIL1, SNAIL2, TWIST1, TWIST2, ZEB1 and ZEB2 [28] (Figure 3B).

Moreover, immunofluorescence analysis showed that N-cadherin was lost from cell-cell contacts, and E-cadherin expression was stronger in AD-ARHGDIA-infected cells (Figure 3C). Then, we transfected AD-GFP and AD-ARHGDIA into epithelial-like HeLa cells and T98G cells and observed that overexpression of ARHGDIA inhibited EMT-like morphological features, such as a spindle-shaped appearance of HeLa cells, and reversed the mesenchymal-like characteristics of glioma cells (Figure 3D). Western blotting analysis was also performed to detect the expression of EMT-related genes in T98G cells overexpressing ARHGDIA or control cells. Collectively, these data suggest that ARHGDIA is an enforcer of the epithelial phenotype and an inhibitor of EMT in gliomas.

### ARHGDIA is a target mRNA of PCBP2 and is also a target of miR-151-5p/miR-16 in gliomas

**Figure 4:**
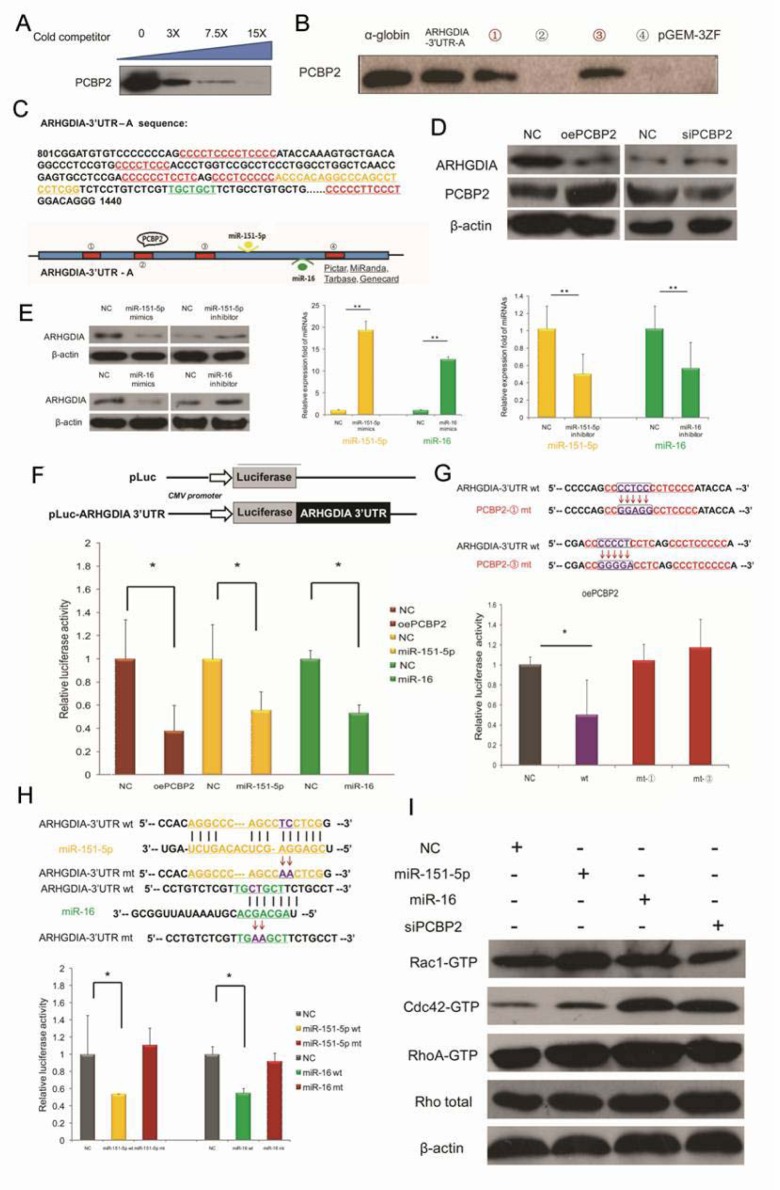
ARHGDIA is a target mRNA of PCBP2 and is also a target of miR-151-5p/miR-16 in gliomas **A**. A specific association between PCBP2 and ARHGDIA-3′UTR-A mRNA was confirmed by a competition assay. Threefold, 7.5-fold or 15-fold of unlabeled RNA was added to compete with the biotin-labeled RNA for interaction with PCBP2. **B**. PCBP2 was detected in the biotin pull-down analysis of complexes formed *in vitro* using 4 biotin-labeled ARHGDIA-3′UTR-A mRNA segments (① - ④) and T98G whole cell lysates. The α-globin-3′UTR and a nonsense sequence were included as the positive and negative controls, respectively. **C**. Nucleotide sequence of ARHGDIA-3′UTR-A from +801 to +1440. The 4 CU-rich patches of potential binding sites for PCBP2 and the positions of the binding sites for miR-151-5p and miR-16 are marked, respectively, in red, orange and green. A schematic representation of the ARHGDIA-3′UTR-A is shown below. **D**. Western blotting analysis showed the protein levels of ARHGDIA and PCBP2 after transfection with NC siRNA and PCBP2 siRNA, control pcDNA3.1 plasmid and PCBP2-pcDNA3.1 (oePCBP2), respectively. β-actin served as an internal control. **E**. Western blotting analysis showed the protein levels of ARHGDIA (left). Real-time PCR analysis of miR-151-5p/miR-16 levels after transfection with synthetic NC mimics and miR-151-5p/miR-16 mimics (middle), synthetic NC inhibitor and miR-151-5p/miR-16 inhibitor (right) in T98G cells, respectively. U6 snRNA was used as an internal control. *P<0.05, **P<0.01 by Student's *t-*test. **F**. Luciferase reporter plasmids containing wild-type ARHGDIA-3′UTR were co-transfected with synthetic NC and miR-151-5p/miR-16 mimics, as well as NC plasmid and oePCBP2 in T98G cells. After 24 h, the cells were harvested, and the normalized luciferase activity was determined. Relative luciferase activity is expressed as the ratio between firefly luciferase and *Renilla* control, adjusted to 100%. (**G**., **H**.) Luciferase assays performed as in F, with luciferase reporter plasmids containing wild-type ARHGDIA-3′ UTR (wt) coupled to the ARHGDIA-3′ UTR mutated for PCBP2 binding CU-rich patches-① (mt-①, ③ (mt-③) (G) and the miR-151-5p/miR-16 binding site mutation (miR-151-5p mt/miR-16 mt) (H). *P<0.05, **P<0.01 by Student's *t-*test. **I**. The activities and protein levels of Rac1, Cdc42, and RhoA GTPases and total Rho protein in T98G cells were determined after transfection with NC mimics, miR-151-5p/miR-16 mimics, and PCBP2 siRNA.

In our previous work [16], we have demonstrated that ARHGDIA is a target mRNA of the RNA-binding protein PCBP2 in gliomas, on the basis of RIP-Chip data and biotin pull-down assays. A specific association between PCBP2 and the ARHGDIA-3′UTR-A mRNA was confirmed by a competition assay (Figure 4A). To identify the exact binding sites, PCBP2 was detected in the biotin pull-down analysis of complexes formed *in vitro* using 4 biotin-labeled ARHGDIA-3′UTR-A mRNA segments (schematic diagram shown in Figure 4C, red panel) and T98G whole cell lysates. The α-globin-3′UTR and a nonsense sequence were included as the positive and negative controls, respectively. The results showed that PCBP2 specifically interacted with ARHGDIA-3′UTR-A in segments ① and ③ (Figure 4B). It is known that the key regulators of mRNA 3′UTRs are miRNAs and RBPs. Database searches using miRNA target prediction programs were performed with the 3′UTR of ARHGDIA, and a putative miR-16 binding site, which is highly conserved among mammals, was predicted by different algorithms; additionally, miR-151-5p has been reported to directly target the 3′UTR of ARHGDIA [7]. The nucleotide sequence of ARHGDIA-3′UTR-A from +801 to +1440 is shown as a schematic diagram in Figure 4C, and the four PCBP2 potential binding sites with CU-rich patches and the positions of the binding sites for miR-151-5p and miR-16 are marked, respectively in red, yellow and green. Western blotting analysis and luciferase reporter assays after overexpression and knockdown of PCBP2 showed that PCBP2 directly interacted with the 3′UTR of ARHGDIA (Figure 4D, 4F).

Luciferase assays with a construct coupled to the ARHGDIA-3′UTR mutated for the two CU-rich patches ① and ③ showed no decrease in luciferase activities compared with that of wild-type after overexpression of PCBP2 (Figure 4G). In addition, inactivating mutations in the miR-151-5p and miR-16 target sites did not decrease the relative luciferase activities as sharply as they did in wild-type counterparts (Figure 4H). Given that both PCBP2 and miR-151/miR-16 target ARHGDIA, which is known to be the negative regulator of Rho GTPases such as Rac1, Cdc42 and Rho, we measured the activities of Rac1, Cdc42 and Rho in T98G cells. Our results showed that increased miR-151-5p and miR-16 expression enhanced Rac1, Cdc42 and RhoA activation. In contrast, knockdown of PCBP2 decreased the activities of Rac1 but not Cdc42 and RhoA in T98G cells, whereas the protein levels of these Rho GTPases were unaltered (Figure 4I).

### Knockdown of PCBP2 inhibits glioma cell migration and invasion via ARHGDIA

**Figure 5:**
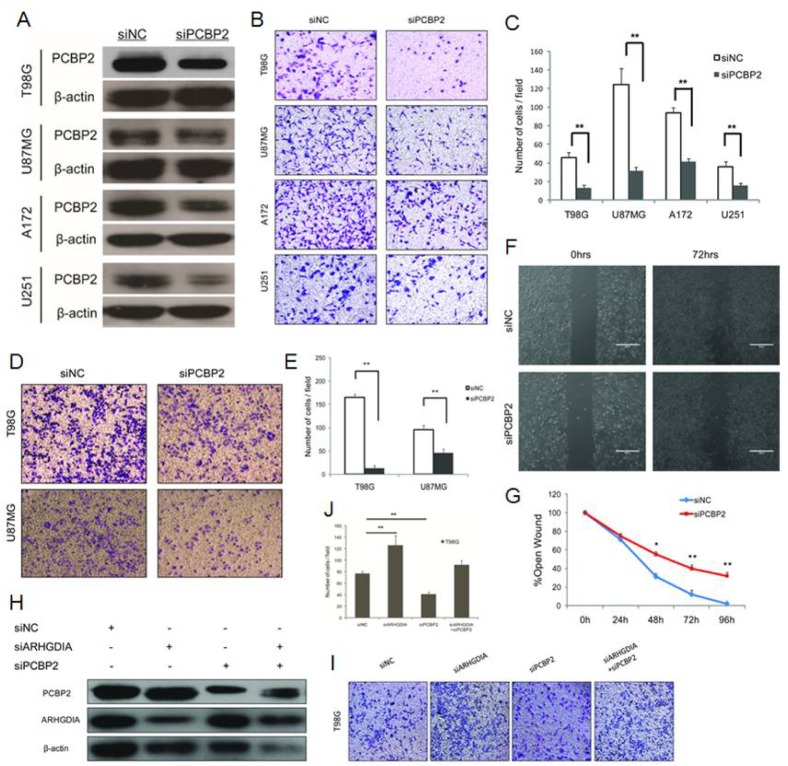
Knockdown of PCBP2 inhibits glioma cell migration and invasion **A**. Western blotting of PCBP2 expression in 4 glioma cell lines (T98G, U87 MG, A172, U251) transfected with NC siRNA and PCBP2 siRNA for 48 h. (**B**., **C.**) Transwell migration assays of 4 glioma cell lines (T98G, U87 MG, A172, U251) were performed after transfection with NC siRNA and PCBP2 siRNA for 48-72 h. The results are representative of at least three independent experiments. Graphs indicate the average number of cells per field of the indicated cell lines in migration assays. Data show the mean ± SD, *P<0.05,**P<0.01. (**D**., **E**.) Invasion assays of 2 glioma cell lines (T98G, U87 MG) were performed after transfection with NC siRNA and PCBP2 siRNA for 48-72 h. The results are representative of at least three independent experiments. Graphs indicate the average number of cells per field of the indicated cell lines in invasion assays. (**F**., **G**.) Wound-healing assays in T98G cells were performed after transfection with NC siRNA and PCBP2 siRNA for 0-96 h. Pictures were taken every 24 h. Graph represents the width of the remaining open wound calculated in relation to time 0. **H**. Co-knockdown of ARHGDIA and PCBP2 for a rescue study. Western blotting analysis revealed that ARHGDIA siRNA and PCBP2 siRNA inhibit both of their targeted proteins after transfection with ARHGDIA siRNA and PCBP2 siRNA individually or co-transfection into T98G cells. (**I**., **J**.) The transwell assays were performed after transfection with ARHGDIA siRNA and PCBP2 siRNA individually or co-transfection into T98G cells. Graphs indicate the average number of cells per field of the indicated cell lines in migration assays. Data show the mean ± SD, *P<0.05,**P<0.01.

Our results demonstrated that PCBP2 directly interacted with the 3′UTR of ARHGDIA, as determined through a series of assays (Figure 4), and we also found that the protein levels of ARHGDIA and PCBP2 were negatively correlated (Figure 1G). Next, we investigated whether PCBP2 affects the glioma migration and invasion via ARHGDIA. We used an effective siRNA that was specifically targeted to PCBP2 and a control siRNA, as previously reported [16] (Figure 5A). After transfection with NC siRNA and PCBP2 siRNA for 48 hours in the same 4 glioma cell lines, transwell migration assays, matrix invasion assays and wound-healing assays were performed. The results indicated that knockdown of PCBP2 inhibited glioma cell migration and invasion *in vitro* (Figure 5B-5G).

To further test our hypothesis, we performed a co-knockdown of ARHGDIA and PCBP2 for a rescue study. Western blotting analysis revealed the depletion of both ARHGDIA and PCBP2 after transfection with ARHGDIA siRNA [7] and PCBP2 siRNA individually into T98G cells. Then, ARHGDIA siRNA and PCBP2 siRNA were co-transfected into T98G cells, thus resulting in partial recovery of both ARHGDIA and PCBP2 depletion (Figure 5H). The transwell assays also demonstrated that the number of migrating cells after the siRNA co-transfection was closer to that of the control than that of ARHGDIA siRNA or PCBP2 siRNA alone (Figure 5I-5J). The rescue study suggested that PCBP2 influences glioma migration and invasion directly through ARHGDIA.

Consistently with the results of the overexpression of ARHGDIA, knockdown of PCBP2 also reversed the mesenchymal-like characteristics of glioma cells. The western blotting analysis showed increases in epithelial marker E-cadherin levels and reductions in the levels of the mesenchymal markers N-cadherin and vimentin, as well as reductions in EMT-related transcription factors, such as Snail1 and Twist1, after transfection with PCBP2 siRNA for approximately 48 h. Moreover, immunofluorescence analysis showed that E-cadherin expression was stronger and N-cadherin was reduced in PCBP2 siRNA-transfected cells. We also found that enforced downregulation of PCBP2 in T98G cells downregulated key transcriptional factors, such as SNAIL1, SNAIL2, TWIST1, TWIST2, ZEB1 and ZEB2 (Supplementary Figure 1).

### miR-151-5p and miR-16 promote glioma cell migration and invasion

To explore the function of miR-151-5p/miR-16 in gliomas, we first examined the expression of miR-151-5p/miR-16 in the 72 glioma tissues compared with 6 control brain tissues. The real-time PCR results showed that both miR-151-5p and miR-16 levels were relatively higher in grade IV glioma tissues than in grade II and grade III glioma tissues compared with control brain tissues (Supplementary Figure 2A, 2B).To determine whether enhanced expression of miR-151-5p and miR-16 could promote glioma cell migration and invasion, transwell migration assays, matrix invasion assays, and wound-healing assays were again carried out after transfection with control NC, miR-151-5p and miR-16 mimics. The results demonstrated that exogenous expression of miR-151-5p and miR-16 promote both migration and invasion of glioma cells (Supplementary Figure 2C-2H).

To assess whether ARHGDIA is the direct functional mediator of miR-151-5p/miR-16-induced glioma cell migration and invasion, we designed the co-knockdown rescue experiments shown in Supplementary Figure 2. Western blotting analysis and transwell assays demonstrated that both the protein level of ARHGDIA and the number of migrating cells after the co-transfection of ARHGDIA siRNA and miR-151-5p/miR-16 inhibitors (anti-miR-151-5p/anti-miR-16) were closer to that of the control level than after individual transfection of miR-151-5p/miR-16 inhibitors Knockdown of ARHGDIA rescued the inhibitory effects of miR-151-5p/miR-16 inhibitors on glioma cell migration. These findings indicated that ARHGDIA is indeed a functional target for miR-151-5p/miR-16, similarly to PCBP2 (Supplementary Figure 3).

### PCBP2 facilitates the binding of miR-151-5p and miR-16 on ARHGDIA

**Figure 6:**
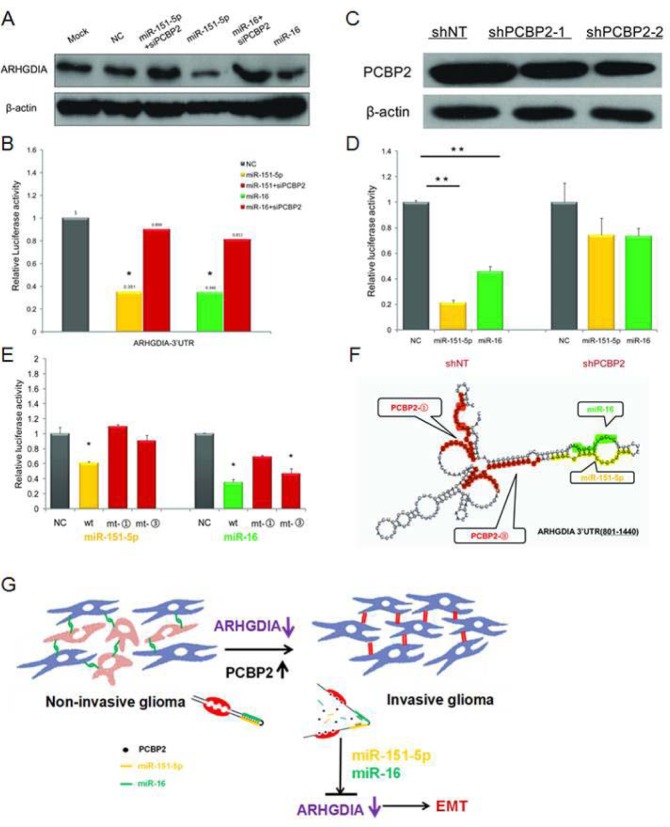
PCBP2 facilitates the function of miR-151-5p and miR-16 on ARHGDIA **A**. Western blotting analysis of ARHGDIA after co-transfection with PCBP siRNA and miR-151-5p/miR-16 mimics compared to individual transfections with miR-151-5p/miR-16 mimics. **B**. Luciferase assays were performed in T98G cells after co-transfection with PCBP siRNA and miR-151-5p/miR-16 mimics compared to individual transfection with miR-151-5p/miR-16 mimics. miR-151-5p/miR-16 and PCBP2 interacted with the wild-type ARHGDIA-3′UTR. Relative luciferase activity is the ratio between firefly luciferase and *Renilla* control, adjusted to 100%. Data show the mean ± SD, *P<0.05,**P<0.01. **C**. The shNT-U87 MG and shPCBP2-U87 MG stable transfectants were used to perform the following luciferase assays. Western blotting analysis of PCBP2 in two shPCBP2-U87 MG cell lines compared to one shNT-U87 MG control cell line harvested at 48-72 h. **D**. Luciferase reporter plasmids containing wild-type ARHGDIA-3′ UTR were co-transfected with synthetic NC and miR-151-5p/miR-16 mimics in the shNT-U87 MG and shPCBP2-U87 MG cell lines. **E**. Luciferase assays were performed in T98G cell lines with ARHGDIA-3′ UTR- wild-type (wt), ARHGDIA -3′ UTR-mt-③ (mt-③), and ARHGDIA -3′ UTR-mt-③ (mt-③) and co-transfected with synthetic NC and miR-151-5p/miR-16 mimics, respectively. **F**. Schematic representation of the conformation of a region of the ARHGDIA-3′UTR-A (+801 to +1440) containing 4 CU-rich patches (including patch ① and ③), a miR-151-5p and a miR-16 binding site, as predicted by RNAfold software, are marked, respectively, in red, orange and green. **G**. Schematic model for the function of ARHGDIA in glioma migration and invasion.

Both PCBP2 and miR-151-5p/miR-16 inhibited ARHGDIA expression. Interestingly, the ARHGDIA-3′UTR-A harbors two evolutionarily conserved PCBP2 recognition elements (the CU-rich patches ① and ③), which have been shown to specifically interact with PCBP2 and are located close to the miR-151-5p and miR-16 target sites (Figure 4C). We sought to elucidate the interaction between PCBP2 and miR-151-5p/miR-16 in promoting the motility and invasiveness of glioma cells through downregulating ARHGDIA. Therefore, we examined the effect of PCBP2 on miR-151-5p/miR-16-induced repression of ARHGDIA by western blotting after co-transfection with PCBP siRNA and miR-151-5p/miR-16 mimics compared to individual transfection with miR-151-5p/miR-16 mimics. The results showed that the ARHGDIA protein level was restored after cells were co-transfected with PCBP siRNA and miR-151-5p/miR-16 mimics compared to cells individually transfected with miR-151-5p/miR-16 mimics (Figure 6A). The luciferase reporter assay was also performed with the 3′UTR of ARHGDIA in T98G cells, which had high endogenous expression of PCBP2. After knockdown of PCBP2 with siRNA, the miR-151-5p and miR-16 suppression of ARHGDIA was compromised (Figure 6B).

To examine the function of miR-151-5p/miR-16 on ARHGDIA in PCBP2 knockout cell lines, we used the shNT-U87 MG and shPCBP2-U87 MG stably transfected cell lines as previously reported [16] to repeat the luciferase assays. The results showed that miR-151-5p/miR-16-induced repression of ARHGDIA was compromised in shPCBP2-U87 MG cells compared with shNT-U87 MG cells (Figure 6C, 6D). In addition, inactivating mutations in the CU-rich patches ① and ③ target sites in ARHGDIA-3′UTR-A of PCBP2 also resulted in a loss of the miR-151-5p and miR-16 suppression effect on ARHGDIA-Luc luciferase activity, although the mutation in the CU-rich patches ③ did not suppress the function of miR-16 (Figure 6E). The fact that PCBP2 knockdown abolished miR-151-5p/miR-16 function, but the loss of its binding sites on the ARHGDIA-3′UTR did not, suggests that PCBP2-induced changes in mRNA structure are involved in regulating miR-151-5p/miR-16 function. Using the secondary structure prediction software RNAfold (Vienna RNA package version 1.8.3), we noticed that the CU-rich patches and the miR-151-5p and miR-16 target sites could form a stem-loop structure with a high base-pair probability (Figure 6F).

Together, these results first indicate that both PCBP2 and miR-151-5p/miR-16 inhibit ARHGDIA expression post-transcriptionally through sites in the ARHGDIA-3′UTR, and second, that efficient suppression of ARHGDIA expression by miR-151-5p/miR-16 requires PCBP2. PCBP2 facilitates the action of miR-151-5p and miR-16 on ARHGDIA through specific mechanisms. In conclusion, our results provide evidence in support of a model in which the high expression of PCBP2 and its binding to ARHGDIA may induce a local change in RNA structure that favors association with miR-151-5p and miR-16, thus leading to suppression of ARHGDIA expression, and the low expression or loss of ARHGDIA induces EMT and promotes glioma migration and invasion (Figure 6G).

## DISCUSSION

Gliomas, especially glioblastoma multiforme, are often described as systemic CNS diseases with a high risk of invasive local recurrence despite surgery, irradiation, and chemotherapy. Generally, distant metastasis or the extraneural metastatic spread of glioma is rare, but the case reports of intracerebral metastases and extraneural metastases of gliomas have been reviewed by Jezewski(29) and Beauchesne [30], respectively. Tumor dissemination and metastatic behavior account for the majority of cancer-associated mortality. Epithelial tumors achieve this progressive state via epithelial-to-mesenchymal transition (EMT) [31,32]. The current research has shown EMT-like changes in malignant gliomas, including a role for SNAIL1/SNAIL2, TWIST1/ TWIST2, ZEB1/ZEB2 and miRNAs as inducers for a cell-invasive phenotype in GBMs [14, 33].

Remodeling of actin filaments into stress fibers is thought to depend on signaling pathways mediated by members of the Rho GTPase family [34]. Rho, Rac, and Cdc42 GTPases regulate many actin-based cytoskeletal processes, including cell adhesion, migration, phagocytosis, cell survival, and contractility [35]. RhoGDIs were initially characterized as simply Rho GTPase inhibitors. Reyes *et al.* have found that αvβ8 integrin interacts with RhoGDI1 and consequently stimulates Rac1 and Cdc42 activation and drives glioblastoma cell invasion [36]. In this report, we show that the *expression* of ARHGDIA is *frequently decreased* in high-grade malignant gliomas. We demonstrated a role for ARHGDIA as a putative metastasis suppressor through analyses of various *in vitro* and *in vivo* models of EMT and metastasis. However, in addition to the functional verification of ARHGDIA, we notably uncovered the relationship between miRNAs and RBPs in modulating the expression and function of a common target gene in glioma migration and invasion.

miRNAs are involved in normal development and in cancer, mainly by associating with 3′ UTRs of messenger RNAs and regulating their expression [37]. We demonstrated that ARHGDIA is a potential target of miR-151-5p and miR-16 in gliomas. Xianghuo *et al.* have shown that miR-151-5p, which is often expressed together with its host gene FAK, significantly increases HCC cell migration and invasion *in vitro* and *in vivo*. miR-151-5p directly targets ARHGDIA, which is also a putative metastasis suppressor in HCC, thus leading to the activation of Rac1, Cdc42 and Rho GTPases [7]. However, the studies of miR-16 in gliomas or cancer invasion are contradictory. Yang *et al.* have shown that miRNA-16 inhibits glioma cell growth and invasion through suppression of BCL2 and the nuclear factor-κB1/MMP9 signaling pathway or possibly through one of the putative target genes, Zyxin [38-40]. Musumeci *et al.* have found a molecular circuitry in which miR-15 and miR-16 and their correlated targets cooperate to promote tumor expansion and invasiveness through the concurrent activity on stromal and cancer cells [41-43]. Our results demonstrated that exogenous expression of miR-151-5p and miR-16 promotes both migration and invasion of glioma cells.

Similarly to miRNAs, RBPs interacts with 3′ UTRs in a sequence-specific manner and both stimulates and inhibits gene expression [44]. For example, Bhattacharyya *et al.* have shown showed that the miR-122-mediated repression of CAT-1 translation is reversed by binding of HuR to a shared site on the AU-rich CAT-1 3′UTR [45]. The RBP HuR reduces c-Myc expression by associating with the c-Myc 3′UTR adjacent to a miRNA let-7-binding site. Lowering HuR or let-7 levels relieves the translational repression of c-Myc, indicating that HuR and let-7 represses c-Myc through an interdependent mechanism [46]. Yujing *et al.* have demonstrated that PCBP2 binds to miRNA precursors and promotes the processing of miRNAs through associating with Dicer by the modulation of cytosolic iron, which can alter the processing of miRNA precursors [47]. Restoration of miR-328 expression rescues the differentiation and survival of leukemic blasts by interacting with PCBP2, promoting release of target mRNA CEBPA from PCBP2-mediated translational inhibition, which is independent of the miRNA seed sequence [48]. Our results revealed that RBP PCBP2 binds to the ARHGDIA-3′UTR, thus inducing a local change in RNA structure that favors association with miR-151-5p/miR-16 and efficiently suppressing ARHGDIA expression. We therefore uncovered a novel RBP-induced structural switch modulating regulation of miRNA-mediated gene expression.

Together, our analyses show that the protein level of ARHGDIA, which is known to be a negative regulator of Rho GTPases such as Rac1, Cdc42 and Rho, is decreased in glioma tumors compared with control brain tissues. We further delineated the role of ARHGDIA as a suppressor regulating EMT and metastasis. Because the protein levels of ARHGDIA and PCBP2 were negatively correlated, combined ARHGDIA and PCBP2 levels may be considered as potential clinical diagnosis biomarkers or may be predictive of outcomes of malignant glioma patients after surgery. Our results also provide evidence supporting a model in which high-grade malignant gliomas, with increased PCBP2 levels, bind the ARHGDIA-3′UTR as well as miR-151-5p/miR-16, thereby inducing a conformational change in the ARHGDIA-3′UTR (Figure 6F). These changes permit more efficient specific binding of miR-151-5p/miR-16 to their target sites on the ARHGDIA-3′UTR. Our results provide strong support for an RBP that modulates miRNA activity by inducing a local structural switch in mRNA.

## MATERIALS AND METHODS

### Cell lines

Human astrocyte cells (HA-c and HA-sp), glioma cell lines (T98G, U87 MG, A172), and HeLa cells were purchased from American Type Culture Collection (ATCC) and grown in Dulbecco's modified Eagle's medium (DMEM) supplemented with 10% fetal calf serum (Invitrogen) plus antibiotics. The U251 cell line (from the Cell Center of Peking Union Medical College) was cultured in minimum essential medium and Iscove's modified Dulbecco's medium supplemented with 10% FBS. The NHA cell line was purchased from the Lonza group and cultured with Clonetics medium and reagents. The other HA cell line was purchased from ScienCell Research Laboratories and cultured with astrocyte medium (catalog 1801). Cell lines were authenticated using STR profile analysis (ATCC) and various tests including cell viability analysis, cytogenetic analyses, flow cytometry and immunohistochemistry. Cell lines were not cultured for more than 6 months before the work described here was conducted.

### Tumor tissues

We included 72 glioma tissues samples and 6 control brain tissues (from the Department of Neurosurgery, Beijing Tiantan Hospital). Malignancy grade (12 samples were grade II, 12 samples were grade III and 48 samples were grade IV) was defined according to the guidelines of the World Health Organization (WHO) (listed in Supplementary Table 1).

### Recombinant adenovirus, plasmids, and siRNAs

The *recombinant adenoviruses* carrying the ARHGDIA-pcDNA3.1/GFP-pcDNA3.1 vectors were packaged by Shanghai *GeneChem* Co., Ltd. (Shanghai, China). The cDNA target sequences of siRNAs for ARHGDIA have been described previously [7], and the cDNA target sequences of siRNAs for PCBP2 have been used in our lab previously(16). All plasmids were purified using the EndoFree Plasmid Maxi Kit (QIAGEN) and transfected into T98G cells using VigoFect (Vigorous Biotechnology). siRNAs were synthesized by the Shanghai GenePharma Company and transfected into glioma cell lines using Lipofectamine 2000 (Invitrogen) at a final concentration of 100 to 200 nM.

### Western blotting

Whole cell extracts were obtained by lysing cells in TNTE buffer (50 mM Tris, pH 7.4, 150 mM NaCl, 1 mM EDTA, 10 mM sodium pyrophosphate, 0.5% Triton X-100, 1 mM sodium vanadate, and 25 mM sodium fluoride) containing protease inhibitors (5 μg/ml PMSF, 0.5 μg/ml leupeptin, 0.7 μg/ml pepstatin, and 0.5 μg/ml aprotinin). The detailed western blotting procedures have been described previously [25]. The protein samples were analyzed using monoclonal mouse anti-ARHGDIA (Santa Cruz Biotechnology, 1:5000), polyclonal rabbit anti-PCBP2 (Beijing Aviva Systems Biology, 1:2000), polyclonal rabbit anti-E-cadherin (Cell Signaling, 1:1000), polyclonal mouse anti-N-cadherin (BD Biosciences, 1:1000), polyclonal mouse anti-vimentin (Santa Cruz Biotechnology, 1:2000), polyclonal rabbit anti-Snail1 (Santa Cruz Biotechnology, 1:2000), polyclonal rabbit anti-Twist1 (Santa Cruz Biotechnology, 1:2000), mouse anti-β-actin (Sigma-Aldrich,1:5000) antibodies. The Quantity One software (Bio-Rad Laboratories) was used to quantify the relative expression levels of the target proteins.

### RNA extraction and quantitative real-time PCR

Total RNA was isolated from treated cells with the TRIzol reagent (Invitrogen) and was reverse transcribed using a reverse transcription system to generate cDNA template according to the manufacturer's instructions. Real-time quantitative PCR was performed using a SYBR-green-containing PCR kit (TaKaRa) and the IQ5 sequence detection system (Applied Biosystems) according to the manufacturer's instructions. To determine relative gene expression, RNA input was normalized to the level of human *GAPDH* mRNA and U6 snRNA (for miRNAs detection). The primer sequences used for RT-PCR and qRT-PCR are listed in Supplementary Table 2.

### Immunohistochemistry and immunofluorescence microscopy

Immunohistochemical analyses of ARHGDIA were conducted using paraffin section specimens of low-grade glioma (n=16) and high-grade-glioma (n=19) tissues from 35 patients (from the Department of Neurosurgery, Beijing Tiantan Hospital). The sections were incubated overnight at 4°C with anti-ARHGDIA antibodies. Staining was performed using a diaminobenzidine staining kit (Zhongshan). For immunocytochemistry, cells were seeded onto glass coverslips and were processed as previously described [26]. DAPI was used to stain nuclei.

### Transwell migration assay, Boyden chamber invasion assay and scratch wound-healing assay

For the transwell migration assay, T98G, U87 MG, U251 and A172 cell lines were used, and 5×10^4^ cells were plated on 8-μm Transwell filters (Corning). For the invasion assays, 1×10^5^ cells were added to the upper chamber of each insert coated with 150 μg Matrigel (BD Biosciences) using the T98G and U87 MG cell lines. The cells were induced to migrate toward medium containing 20% FBS for 24 h (for migration assay) and 48 h (invasion assay) in the CO_2_ incubator. Non-invading cells were removed with a cotton swab. The remaining cells were fixed and stained in dye solution containing 0.1% crystal violet and 20% methanol. The cells that had migrated or invaded were counted and imaged using an IX71 inverted microscope (Olympus Corp.). Ten random fields were chosen, and cell numbers were averaged. Transwell chambers were separated by membranes with 12-μm pores, and FBM medium containing 500 ng/ml prolactin was placed in the lower chamber. For scratch wound-healing assays, when the transfected/infected glioma cells reached 80% confluence, a wound was created by scratching with a 200 μl pipette tip. After scratching, the detached cells were removed by washing twice and then the cells were maintained in fresh medium. Cells were imaged at 0 h, 24 h, 48 h, 72 h and 96 h after scratching, and the migration distance was calculated by measuring the width of the wound.

### miRNA target prediction

To identify potential miRNA binding sites within the 3′UTR of ARHGDIA, the following bioinformatics databases were used: TargetScan (http://www.targetscan.org/), PicTar (http://pictar.mdc-berlin.de/), miRanda (http://www.microrna.org/), Tarbase (http://www.hsls.pitt.edu/), Genecard (http://www.genecards.org/).

### Dual-luciferase reporter assays

ARHGDIA (NM_004309) human cDNA clone was amplified and cloned into pRluc with XhoI and XbaI. T98G cells (5×10^4^) were seeded in 24-well plates 24 h before transfection. The following day, 30-100 ng of the *ARHGDIA* promoter reporter plasmid (WT or MT) along with 5 pmol of NC (negative control), miR-151-5p or miR-16, and 200 ng of the internal control plasmid constitutively expressing *Renilla* luciferase was co-transfected using Lipofectamine 2000 (Invitrogen). Cells were collected 24 h post-transfection and assayed for luciferase activity using a Glomax 96 microplate luminometer (Promega). For each experimental trial, cells were transfected with the same plasmids in quadruplicate, and each well was assayed using the dual-luciferase reporter assay system. Firefly luciferase activity was normalized with Renilla luciferase activity for each transfected well. These experiments were repeated more than three times, and the *P* value was calculated with a two-tailed Student's *t* test.

### EMT-related marker genes and transcription factors

The expression level of the epithelial cell marker E-cadherin and the mesenchymal cell markers N-cadherin and vimentin were detected by western blotting and immunofluorescence microscopy. The expression levels of EMT-related transcription factors were detected by quantitative RT-PCR as described previously [27].

### RNA immunoprecipitation (RIP)

RIP was performed as described in detail previously [28]. Briefly, a tandem affinity-purified tag, biotin acceptor peptide (BAP), was fused to the 5′ terminal of the coding sequences of ARHGDIA and GFP, and they were then co-transfected with BirA (biotin ligase) into T98G cells. Biotin (1 mM) was added to the culture medium immediately after transfection. After 48 hours, the biotin-tagged proteins and RNA complexes were isolated from the cell lysate using high-affinity streptavidin-Sepharose beads (GE Healthcare). RNP was eluted from the beads, and the RNA was purified from RNP using TRIzol (Invitrogen).

### Biotin pull-down assay

Fragments located in the 3′ UTR of ARHGDIA were cloned into a pGEM-3zf (+) vector containing a T7 promoter. The primers are shown in Supplemental Table 2. The biotin-labeled sense RNA probes were synthesized *in vitro* using T7 RNA polymerase (TaKaRa Bio). Cytoplasmic cell extracts were isolated from T98G cells using nuclear protein extraction reagents (Thermo Scientific). RNA affinity capture was subsequently conducted with streptavidin-Sepharose beads as described previously [16].

### Rho GTPase activation assay

The activation of the Rho-family GTPases was determined with RhoA and Rac1/Cdc42 activation assay kits (Upstate Biotechnology) in accordance with the manufacturer's instructions. Analysis was performed by SDS–PAGE and western blotting with anti-RhoA (1:1000), anti-Rac1 (1:1000) and anti-Cdc42 (1:1000) antibodies.

### Xenograft model in nude mice

Four-week-old BALB/c athymic nu/nu mice (Vital River) were injected in the right flank with AD-ARHGDIA-infected and in the left flank with AD-GFP-infected U87 MG cells (1 × 10^6^ cells in 100 μl physiological saline). The tumors were measured weekly or every 5 days thereafter. Tumor volume (V) was estimated using a caliper by measuring the length (L) and width (W), where V = (L × W^2^)/2. The data were analyzed by a two-tailed Student's *t* test. Intracranial orthotopic xenografts were established by implanting 5 ×10^5^ Luc-U87 MG stable cells. The plvx-U87 MG and ARHGDIA-U87 MG stable transfectants were constructed by Shanghai Chempartner. Five- to six-week-old BALB/c athymic nu/nu mice were anesthetized, and the implantation of U87 MG cells into the third ventricle was performed stereotactically (2 mm lateral and 0.5 mm anterior to the bregma; depth 1.5 mm from the dura). Tumor size was quantified by bioluminescence imaging.

### Statistics

Data are presented as the mean ± SD unless otherwise indicated. Statistical significance was determined by a paired or unpaired two-tailed Student's *t* test, and a *P* value of less than 0.05 was considered statistically significant.

### Study approval

All human tissue samples of control brain tissues and gliomas were obtained from the Department of Neurosurgery, Beijing Tiantan Hospital. All samples were classified according to the third edition of the histological grades of tumors of the nervous system published by the WHO in 2000. Informed consent for the use of samples was obtained from all patients before surgery, and approval was obtained from the Medical Ethics Committee of the Beijing Tiantan Hospital (Beijing, China). All animal studies were approved by the IACUC of the Center for Experimental Animal Research (China), and all animal experiments were performed in accordance with institutional guidelines and abided by the declaration of ethical approval for experiments (animal experiment ethical investigation tab ACUC2010A02-123).

## SUPPLEMENTARY MATERIAL FIGURES AND TABLES


