# Supplementary material for: Interplay between PCBP2 and miRNA modulates ARHGDIA expression and function in glioma migration and invasion
Source: Oncotarget. 2016 Jan 9;7(15):19483–98. doi: 10.18632/oncotarget.6869 (PMC4991396; doi:10.18632/oncotarget.6869)
Supplement: Supplementary file 1 [file oncotarget-07-19483-s001.pdf]

# Interplay between PCBP2 and miRNA modulates *ARHGDIA* expression and function in glioma migration and invasion

## Supplementary Material

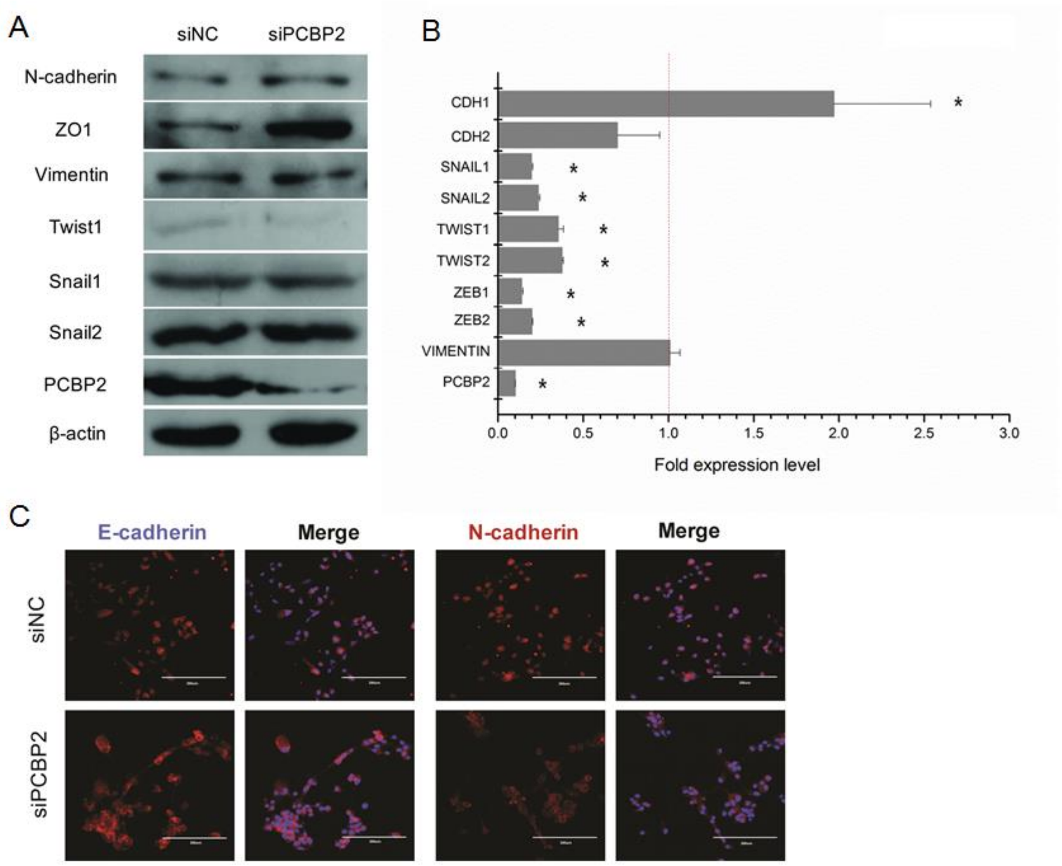


**Supplementary Fig.1** Knockdown of PCBP2 reverses mesenchymal-like characteristics of glioma cells. **(A)** Immunoblot showing the down-regulation of mesenchymal markers and meanwhile up-regulation of epithelial markers after transfection with NC siRNA and PCBP2 siRNA for 48h into T98G cells. **(B)** Quantitative real-time PCR analysis of PCBP2 and EMT-related genes in T98G after transfection with NC siRNA and PCBP2 siRNA.Real-time PCR analysis values were normalized to GAPDH. data show the mean±S.D. *$P<0.05$,**$P<0.01$ by Student's *t*-test. **(C)** Immunofluorescence microscopy analysis of E-cadherin(red) and N-cadherin (red) , merge with nuclear DAPI staining (blue) in NC siRNA and PCBP2 siRNA transfection T98G cells. The scale bars represent 200 µm.

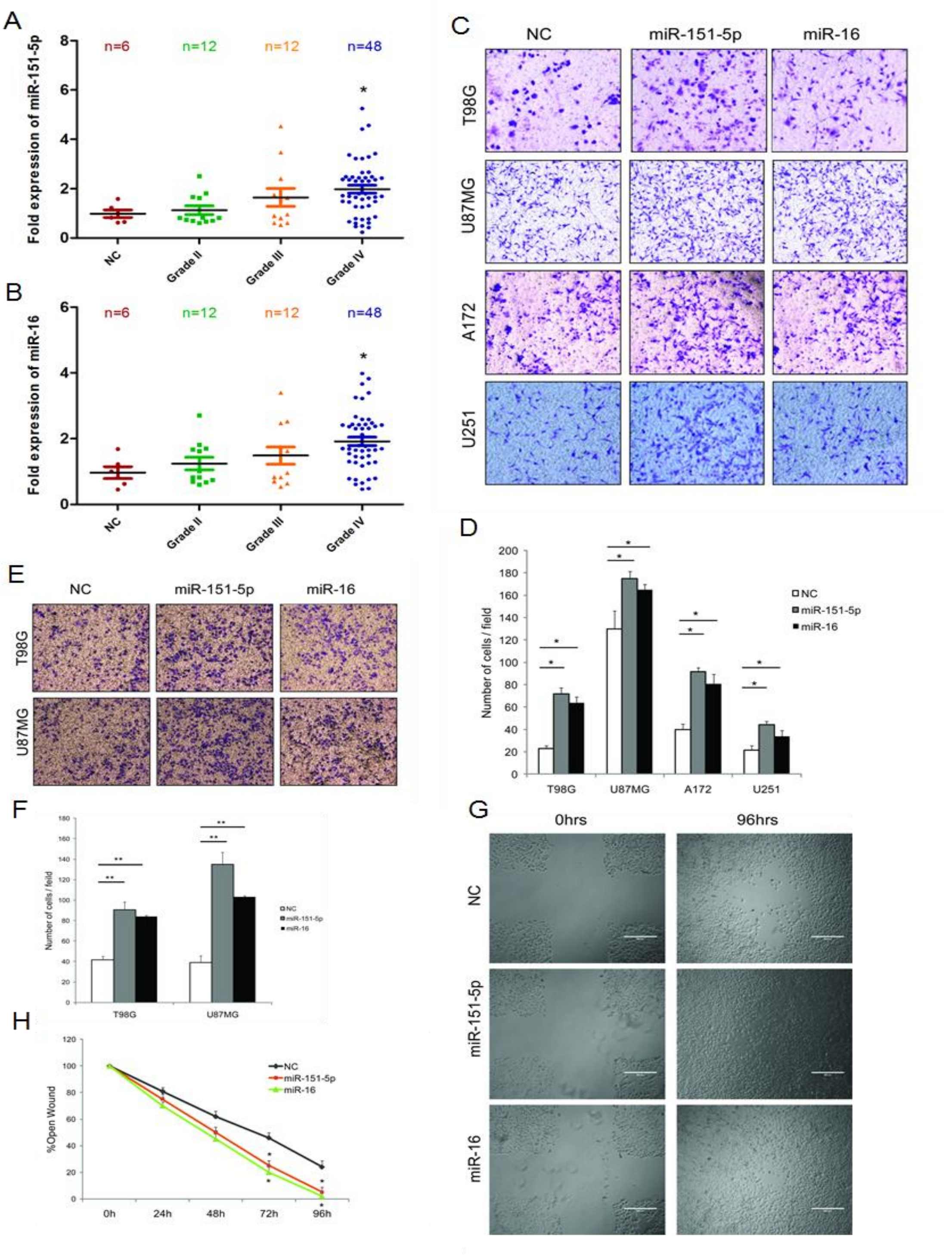


**Supplementary Fig.2** . High expression of miR-151-5p/miR-16 promotes glioma cell migration and invasion (A,B) Relative miR-151-5p and miR-16 expression were determined by TaqMan real-time PCR in in 6 control brain tissues, 12×II grade, 12×III grade, and 48×IV glioma tissues, U6 snRNA was used as a control. (C-F) Transwell migration in 4 glioma cells (T98G, U87 MG, A172, U251) and invasion assays in 2 glioma cells (T98G, U87 MG) were performed after transfection with control NC, miR-151-5p or

miR-16 mimics in 48-72h.The results are representatives of at least three independent experiments. Graphs indicate the average number of cells per field at the indicated cell lines in migration assays. Data show the mean±S.D. *$P < 0.05$,**$P < 0.01$. (G, H) The wound-healing assays in T98G cells transfection with control NC, miR-151-5p and miR-16 mimics for 0-96h. Pictures were taken every 24h. A representative picture was shown on time 0h and 96h.Graph represents the width of the remaining open wound calculated in relation to time 0 separation, *$P < 0.05$, **$P < 0.01$.

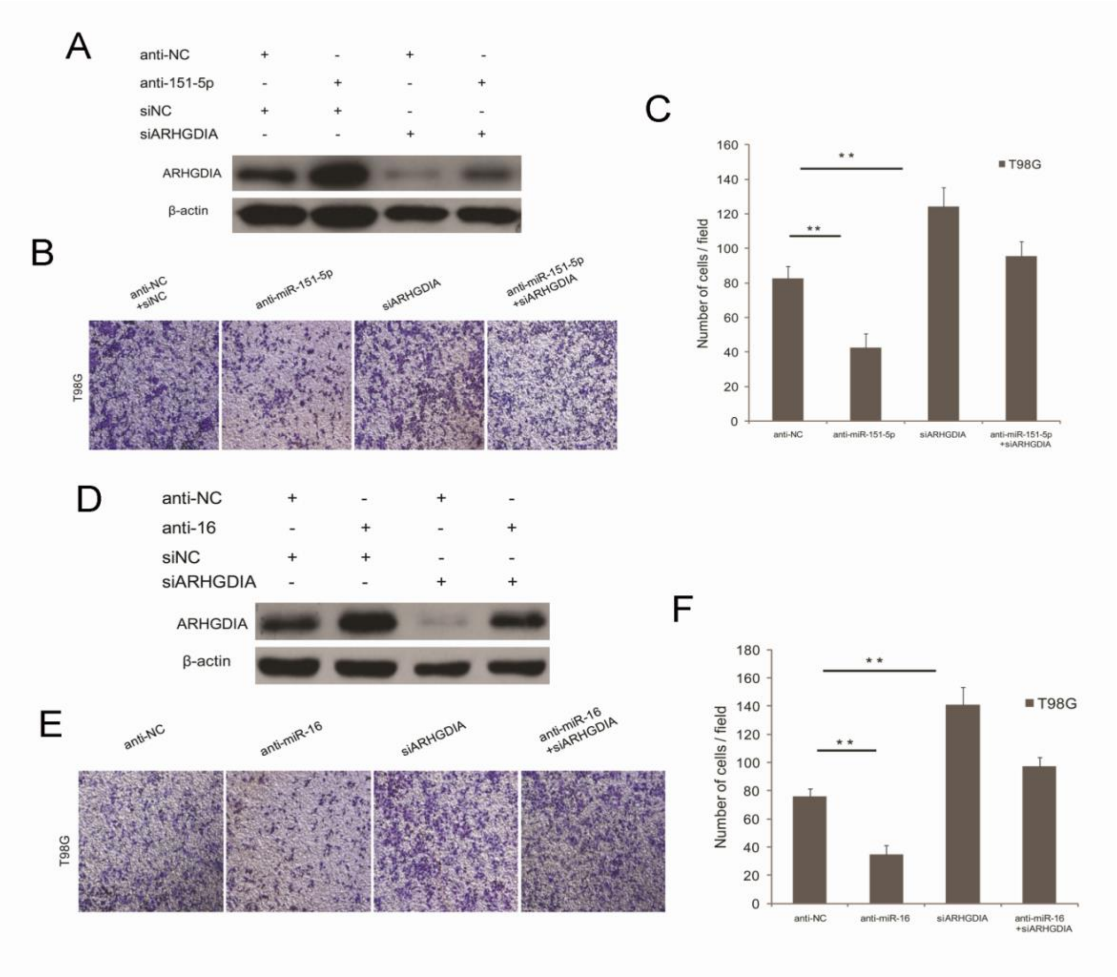


**Supplementary Fig.3** The rescue assays of miR-151-5p and miR-16.**(A-C)** The co-knockdown of ARHGDIA and miR-151-5p for a rescue study. Western blotting and transwell assays after transfection with ARHGDIA siRNA and miR-151-5p inhibitor (anti-miR-151-5p) individually or cotransfected into T98G cells. Graphs indicate the average number of cells per field at the indicated cell lines in migration assays. Data show the mean±S.D. *$P < 0.05$,**$P < 0.01$. **(D-F)** The co-knockdown of ARHGDIA and miR-16 for a rescue study. Western blotting and transwell assays after transfection with ARHGDIA siRNA and miR-16 inhibitor (anti-miR-16) individually or cotransfected into T98G cells. Graphs indicate the average number of cells per field at the indicated cell lines in migration assays. Data show the mean±S.D. *$P < 0.05$,**$P < 0.01$.

**Supplementary Table 1**

Glioma clinical data

| No. | Age | Gender | Grade | Recurrence | Hystotype |
|---|---|---|---|---|---|
| 1 | 22 | M | III | YES | oligo-astrocytoma |
| 2 | 68 | M | IV | NO | glioblastoma multiforme |
| 3 | 49 | M | II | NO | oligodendroglioma |
| 4 | 50 | F | III | YES | glioblastoma multiforme |
| 5 | 48 | M | IV | YES | glioblastoma multiforme |
| 6 | 37 | F | III | NO | oligodendroglioma |
| 7 | 46 | M | IV | NO | glioblastoma multiforme |
| 8 | 57 | F | IV | NO | oligo-astrocytoma |
| 9 | 44 | F | IV | YES | glioblastoma multiforme |
| 10 | 22 | M | IV | YES | glioblastoma multiforme |
| 11 | 44 | M | IV | YES | glioblastoma multiforme |
| 12 | 34 | F | II | NO | diffuse astrocytoma |
| 13 | 36 | F | IV | NO | glioblastoma multiforme |
| 14 | 58 | M | IV | NO | glioblastoma multiforme |
| 15 | 46 | M | IV | NO | glioblastoma multiforme |
| 16 | 47 | F | III | NO | astrocytoma |

| | | | | | |
|---|---|---|---|---|---|
| 17 | 39 | F | IV | NO | anaplastic astrocytoma |
| 18 | 34 | M | III | NO | diffuse oligodendroglioma |
| 20 | 37 | M | III | YES | astrocytoma |
| 21 | 62 | M | IV | NO | glioblastoma multiforme |
| 22 | 28 | F | IV | NO | anaplastic astrocytoma |
| 23 | 43 | M | III | YES | oligo-astrocytoma |
| 24 | 45 | M | IV | YES | glioblastoma multiforme |
| 25 | 35 | M | III | NO | oligodendroglioma |
| 27 | 62 | M | IV | NO | glioblastoma multiforme |
| 28 | 34 | M | IV | YES | glioblastoma multiforme |
| 29 | 39 | M | III | NO | astrocytoma |
| 30 | 42 | M | IV | YES | glioblastoma multiforme |
| 31 | 38 | M | IV | YES | glioblastoma multiforme |
| 32 | 39 | M | IV | YES | glioblastoma multiforme |
| 36 | 43 | F | IV | NO | glioblastoma multiforme |
| 37 | 56 | M | III | NO | oligo-astrocytoma |
| 39 | 30 | F | IV | YES | anaplastic oligo-astrocytoma |
| 41 | 48 | F | IV | YES | glioblastoma multiforme |
| 42 | 34 | M | II | NO | astrocytoma |
| 44 | 60 | M | IV | NO | glioblastoma multiforme |

| | | | | | |
|---|---|---|---|---|---|
| 45 | 76 | M | IV | NO | glioblastoma multiforme |
| 47 | 73 | F | IV | NO | glioblastoma multiforme |
| 49 | 24 | F | IV | NO | glioblastoma multiforme |
| 50 | 29 | M | IV | NO | anaplastic oligo-astrocytoma |
| 51 | 29 | F | II | NO | oligodendroglioma |
| 53 | 22 | F | IV | NO | glioblastoma multiforme |
| 54 | 40 | F | IV | NO | glioblastoma multiforme |
| 55 | 48 | M | III | NO | glioneuronal tumor |
| 56 | 41 | F | II | NO | oligo-astrocytoma |
| 57 | 72 | F | IV | NO | glioblastoma multiforme |
| 59 | 60 | M | IV | NO | anaplastic astrocytoma |
| 60 | 28 | F | IV | YES | glioblastoma multiforme |
| 61 | 50 | F | IV | NO | glioblastoma multiforme |
| 62 | 30 | F | II | NO | astrocytoma |
| 63 | 46 | F | III | NO | anaplastic oligo-astrocytoma |
| 65 | 24 | M | IV | YES | glioblastoma multiforme |
| 66 | 46 | F | II | NO | oligodendroglioma |
| 67 | 42 | F | II | NO | oligo-astrocytoma |
| 69 | 49 | M | IV | NO | glioblastoma multiforme |
| 71 | 45 | F | IV | NO | glioblastoma multiforme |

| | | | | | |
|---|---|---|---|---|---|
| 72 | 53 | M | IV | NO | glioblastoma multiforme |
| 73 | 49 | M | IV | NO | glioblastoma multiforme |
| 74 | 48 | M | III | YES | anaplastic oligodendroglioma |
| 75 | 26 | F | III | NO | oligo-astrocytoma |
| 76 | 70 | M | IV | NO | glioblastoma multiforme |
| 77 | 68 | F | IV | NO | glioblastoma multiforme |
| 78 | 37 | M | III | YES | anaplastic astrocytoma |
| 79 | 40 | M | II | NO | oligodendroglioma |
| 82 | 54 | F | III | NO | anaplastic oligodendroglioma |
| 84 | 55 | M | III | YES | anaplastic oligo-astrocytoma |
| 87 | 28 | F | III | NO | anaplastic astrocytoma |
| 88 | 29 | F | III | NO | oligodendroglioma |
| 90 | 40 | M | II | YES | oligodendroglioma |
| 93 | 69 | F | II | NO | oligodendroglioma |
| 98 | 32 | M | II | NO | oligo-astrocytoma |

**Supplementary Table 2**

| | |
|---|---|
| miRNAs primers | |
| miR-151-5p RT primer | 5'-GTCGTATCCAGTGCAGGGTCCGAGGTATTCGCACTGGATACGACACTAGA-3' |
| miR-16 RT primer | 5'-GTCGTATCCAGTGCAGGGTCCGAGGTATTCGCACTGGATACGACGCGGTT-3' |
| u6 RT primer | 5'-GTCGTATCCAGTGCAGGGTCCGAGGTATTCGCACTGGATACGACCAGGCG-3' |
| miR-16 rltm primer forward | 5'-GCCCCTTCGTCGTTAGA-3' |
| miR-151-5p rltm primer forward | 5'-CGCGTCGAGGAGCTCACA-3' |
| U6 rltm primer forward | 5'-CTGGTTAGTACTTGGACGGGAGAC-3' |
| Universal primer reverse | 5'-GTGCAGGGTCCGAGGT-3' |
| ARHGDIA PCR primers | |
| ARHGDIA 3’UTR forward primer | 5'-CCCTCGAGAGGAGGCACCCAAGGGTAT-3' |
| ARHGDIA 3’UTR reverse primer | 5'-GCTCTAGAAAAGGCAGAGGCAGGACAA-3' |
| ARHGDIA CDS forward primer | 5'-GCGGGGATCCTTGAGCATGGCTGAGCAG-3' |
| ARHGDIA CDS reverse primer | 5'-GCCGCTCGAGGTCCTTCCAGTCCTTCTTGAT-3' |
| ARHGDIA rltm forward primer | 5'-TTTCCGCAGACCCCAACG-3' |
| ARHGDIA rltm reverse primer | 5'-ATCTCTCGGTTAACCCGGAAAG-3' |
| Biotin pull-down probe primers | |
| ARHGDIA-3' UTR-A forward | 5'--GGAATTCGACTGACGGACGGACGAC-3’ |

| | |
|---|---|
| ARHGDIA-3' UTR-A reverse | 5'--CGGGATCCTGTGACGGGGAGATAGAACCT-3' |
| ARHGDIA-3' UTR-A-① forward | 5'-GGAATTCACGGACAGGCGGATGTGT-3' |
| ARHGDIA-3' UTR-A-① reverse | 5'-CCCAAGCTTAGCACTTTGGTATGGGGAGG-3' |
| ARHGDIA-3' UTR-A-② forward | 5'--GGAATTCCATACCAAAGTGCTGACAGGC-3' |
| ARHGDIA-3' UTR-A-② reverse | 5'-CCCAAGCTTTCGGTTGAGCCAGGCCA-3' |
| ARHGDIA-3' UTR-A-③ forward | 5'-GGAATTCTCAACCGAGTGCCTCCGA-3' |
| ARHGDIA-3' UTR-A-③ reverse | 5'-CCCAAGCTTTGGGTGGGGGAGGGCT-3' |
| ARHGDIA-3' UTR-A-④ forward | 5'-GGAATTCGCCTGGCTCAACCGAGTG-3' |
| ARHGDIA-3' UTR-A-④ reverse | 5'-CCCAAGCTTACGAGACAGGAGACCGAGGA-3' |
| α-globin-3' UTR forward | 5'-GGAATTCGCTGGAGCCTCGGTGG-3' |
| α-globin-3' UTR reverse | 5'-CCCAAGCTTGCCGCCCACTCAGACTT-3' |
| mutation primers | |
| miR-151-5p mt forward | 5'-ACCCACAGGCCCAGCCAATCCGGTCTCCT-3' |
| miR-151-5p mt reverse | 5'-TTGGCTGGGCCTGTGGGTGGGGGAGGG-3' |
| miR-16 mt forward | 5'-GTCTCCTGTCTCGTTGAAGCTTCTGCCTGTG-3' |
| miR-16 mt reverse | 5'-TTCAACGAGACAGGAGACCGAGGAGGCTGGG-3' |
| ARHGDIA-3' UTR-A-①-mt forward | 5'-TGTGTCCCCCCCAGCCGGAGGCCTCCCCATACCAAAGTGCT-3' |
| ARHGDIA-3' UTR-A-①-mt reverse | 5'-AGCACTTTGGTATGGGGAGGCCTCCGGCTGGGGGGGACACA-3' |
| ARHGDIA-3' UTR-A-③-mt forward | 5'-AACCGAGTGCCTCCGACCGGGGACCTCAGCCCTCCCCCAC-3' |
| ARHGDIA-3' UTR-A-③-mt reverse | 5'-GTGGGGGAGGGCTGAGGTCCCCGGTCGGAGGCACTCGGTT-3' |

| EMT-related genes primers | |
|---|---|
| CDH1 forward | 5'-GCCCCATCAGGCCTCCGTTT-3' |
| CDH1 reverse | 5'-ACCTTGCCTTCTTTGTCTTTGTTGGA-3' |
| CDH2 forward | 5'-ACAGTGGCCACCTACAAAGG-3' |
| CDH2 reverse | 5'-CCGAGATGGGGTTGATAATG-3' |
| SNAI1 forward | 5'-TGCGCTACTGCTCGGCGAAT-3' |
| SNAI1 reverse | 5'-AGGGCTGCTGGAAGGTAAACTCTGG-3' |
| SNAI2 forward | 5'-CTGGGCGCCCTGAACATGCAT-3' |
| SNAI2 reverse | 5'-GGCTTCTCCCCCGTGTGAGTTCTA-3' |
| TWIST1 forward | 5'-CACGAGCGGCTCAGCTACGC-3' |
| TWIST1 reverse | 5'-ACAATGACATCTAGGTCTCCGGCCC-3' |
| TWIST2 forward | 5'-GGCGCGCCAGGAGGAGATTCT-3' |
| TWIST2 reverse | 5'-TGCTCACTCCCGCCAACGTT-3' |
| ZEB1 forward | 5'-AGTGGTCATGATGAAAATGGAACACCA-3' |
| ZEB1 reverse | 5'-AGGTGTAACTGCACAGGGAGCA-3' |
| ZEB2 forward | 5'-GACAGATCAGCACCAAATGC-3' |
| ZEB2 reverse | 5'-GCTGATGTGCGAACTGTAGG-3' |
| VIMENTIN forward | 5'-GAGAACTTTGCCGTTGAAGC-3' |
| VIMENTIN reverse | 5'-GCTTCCTGTAGGTGGCAATC-3' |
| GAPDH forward | 5'-GGAGTCAACGGATTTGGTCGTA-3' |

| | |
|---|---|
| GAPDH reverse | 5'-GGCAACAATATCCACTTTACCAGAGT-3' |
| PCBP2 siRNA | 5'-GGCCTATACCATTCAAGGA-3' |
| ARHGDIA siRNA | 5'-GGAAAGGCGUCAAGAUUGAdTdT-3' |
